# Glucagon-Like Peptide-1 Receptor Agonist Therapy Reduces Fibrosis-4 Index in Metabolic Dysfunction-Associated Steatotic Liver Disease

**DOI:** 10.14740/gr2136

**Published:** 2026-06-16

**Authors:** Jonathan Weng, Anderson Huang, Tamiru B. Berake, Sarah L. Chen, Brian M. Yan, Raffi Karagozian

**Affiliations:** a Department of Medicine, Tufts Medical Center, Boston, MA, USA; b Division of Gastroenterology and Hepatology, Tufts Medical Center, Boston, MA, USA; c These authors contributed equally to the manuscript.

**Keywords:** GLP-1, FIB-4, MASLD, Liver fibrosis

## Abstract

**Background:**

Metabolic dysfunction-associated steatotic liver disease (MASLD) is a leading contributor to liver-related morbidity and mortality. Glucagon-like peptide-1 receptor agonists (GLP-1 RAs) have shown promise in improving MASLD-related outcomes, but their effect on hepatic fibrosis remains unclear. This study evaluated the impact of GLP-1 RA therapy on liver fibrosis, utilizing the validated fibrosis-4 (FIB-4) index as a noninvasive marker of fibrosis severity.

**Methods:**

In this retrospective cohort study, we identified patients within a large academic health system with MASLD, a baseline FIB-4 ≥ 1.3 (indicating intermediate or higher risk of hepatic fibrosis), and at least two GLP-1 RA prescriptions within a 90-day period between 2020 and 2024. Patients with cirrhosis or alcohol use disorder were excluded. Values were collected at treatment initiation and 12 months later. One-tailed paired *t*-tests assessed differences in FIB-4 and secondary outcomes, and multivariable linear regression identified independent predictors of FIB-4 change.

**Results:**

Among 229 patients with MASLD who were treated with GLP-1 RAs, 29 had a baseline FIB-4 ≥ 1.3. After 12 months, mean FIB-4 decreased by 0.21 from 1.94 to 1.73 (95% confidence interval (CI) −0.38 to −0.05; P = 0.019). Significant reductions were also observed in AST (−15.8 U/L), ALT (−21.9 U/L), body mass index (BMI, −2.6 kg/m^2^), and hemoglobin A1c (A1c, −1.1%; all P < 0.001). On multivariable analysis, baseline FIB-4 index was the strongest predictor of FIB-4 change (β = −0.538; P < 0.001), and FIB-4 improvement was independent of BMI change (β = 0.020; P = 0.243).

**Conclusion:**

In patients with MASLD at intermediate or higher risk of hepatic fibrosis, GLP-1 RA therapy was associated with significant reductions in FIB-4, AST, ALT, BMI, and A1c over 12 months, supporting a potential antifibrotic effect. The FIB-4 index may serve as a practical noninvasive marker for monitoring fibrosis response in MASLD.

## Introduction

Metabolic dysfunction-associated steatotic liver disease (MASLD), previously known as nonalcoholic fatty liver disease (NAFLD), is one of the most prevalent liver disorders worldwide, affecting nearly one-third of the global population [1–3]. Within this spectrum, metabolic dysfunction-associated steatohepatitis (MASH), formerly nonalcoholic steatohepatitis (NASH), is marked by steatosis with hepatocellular injury and inflammation, and occurs in an estimated 20% of individuals with MASLD [4]. The high prevalence, coupled with the potential for severe complications—including progression to cirrhosis and hepatocellular carcinoma—underscores the urgent need for effective therapeutic interventions to halt or reverse disease progression [5].

The clinical burden of MASLD extends well beyond the liver. A growing body of evidence underscores a bidirectional relationship between MASLD and cardiovascular disease (CVD), rooted in shared pathophysiological mechanisms including chronic low-grade inflammation, endothelial dysfunction, insulin resistance, and ectopic lipid accumulation [6–9]. MASLD is independently associated with an elevated risk of coronary artery disease, heart failure with preserved ejection fraction (HFpEF), atrial fibrillation, and all-cause cardiovascular mortality [6–9]. The same hepatic and visceral adipose inflammatory milieu that drives fibrogenesis in MASLD also promotes endothelial injury, oxidative stress, and adverse cardiac remodeling, establishing MASLD as a component of the broader cardiovascular-kidney-metabolic syndrome [8].

Glucagon-like peptide-1 receptor agonists (GLP-1 RAs) are widely used in the management of type 2 diabetes and obesity, where they promote weight reduction, improve insulin sensitivity, and enhance glycemic control [10–12]. Increasing evidence also supports their benefit in MASLD, as these agents reduce liver enzyme levels and hepatic fat content, suggesting a therapeutic role in this population [13–19]. Importantly, GLP-1 RAs have demonstrated benefit across the entire cardiometabolic axis by reducing hepatic fat, improving glycemic control, and lowering cardiovascular event rates, suggesting that hepatoprotective effects observed with this drug class may be mechanistically intertwined with improvements in systemic cardiometabolic health [6–9].

The effect of GLP-1 RA therapy on fibrosis, however, is less clear. Liver fibrosis results from the accumulation of extracellular matrix proteins in response to chronic liver injury, and its severity is a key determinant of long-term outcomes in MASLD [5, 20]. Given the central role of fibrosis in the progression to end-stage liver disease, assessing the antifibrotic potential of GLP-1 RAs is of considerable clinical interest. Noninvasive indices such as the fibrosis-4 (FIB-4) index have been established as useful tools for evaluating fibrosis severity in clinical settings [21–23]. The FIB-4 index is calculated from age, aspartate aminotransferase (AST), alanine aminotransferase (ALT), and platelet count. Current guidance from the American Association for the Study of Liver Diseases (AASLD) recommends secondary risk assessment for patients with FIB-4 ≥ 1.3, corresponding to an intermediate or higher risk of fibrosis, and direct referral to hepatology care for those with FIB-4 > 2.67, which suggests a high risk of clinically significant fibrosis [24].

Emerging data suggest that GLP-1 RA therapy may improve steatohepatitis without worsening fibrosis [14, 25, 26], though presently definitive evidence of fibrosis regression is limited. As GLP-1 RAs become more widely used in patients with metabolic risk factors, understanding their potential role in modulating liver fibrosis could have significant therapeutic implications. This study was designed to address this question by retrospectively evaluating patients with MASLD and baseline intermediate or higher risk of fibrosis, defined by a FIB-4 index ≥ 1.3, who were treated with GLP-1 RAs. By comparing pre- and post-treatment FIB-4 values, along with associated metabolic parameters, we aimed to provide further insight into the potential antifibrotic effects of GLP-1 RAs and to inform future prospective studies in this area.

## Materials and Methods

### Study population

We performed a retrospective cohort study of adult patients with MASLD who were treated with GLP-1 RAs. Patients were identified through the Tufts Research Data Warehouse and the Tufts Medicine electronic health record (EHR), which includes patients receiving care at Tufts Medical Center, Lowell General Hospital, MelroseWakefield Hospital, and Tufts Medicine-affiliated community practices. Inclusion criteria were: 1) age ≥ 18 years at the time of first GLP-1 RA prescription; 2) a diagnosis of MASLD, MASH, NAFLD, nonalcoholic fatty liver (NAFL), or NASH (ICD-10 codes K75.8 or K76.0); 3) a baseline FIB-4 index ≥ 1.30; and 4) documented exposure to a GLP-1 RA (dulaglutide, exenatide, liraglutide, lixisenatide, semaglutide, or tirzepatide), defined as two or more dispensed prescriptions within any 90-day interval between January 1, 2020, and April 30, 2024. Exclusion criteria included a history of alcohol use disorder (ICD-10 code F10) or cirrhosis (ICD-10 code K74).

### Data collection

The index date was defined as the date of the first qualifying GLP-1 RA prescription. Baseline demographic and clinical data, including age, sex, and GLP-1 RA type and indication, were collected at this time. Body mass index (BMI) and laboratory values—AST, ALT, platelet count, hemoglobin A1c (A1c)—were collected at baseline and 12 months after the index date. Baseline values were the closest available measurements within the 6 months prior to the index date, while post-treatment values were the closest available measurements at 12 ± 6 months after the index date. The FIB-4 index was calculated as: (Age (years) × AST (U/L))/(platelets (k/µL) × (ALT (U/L))^0.5^) [21].

### Outcome measures

The primary outcome was the change in FIB-4 index from baseline to 12 months post-treatment. Secondary outcomes included changes in the individual components of the FIB-4 index (AST, ALT, platelet count), BMI, and A1c over the same interval.

### Statistical analysis

Continuous variables were summarized using means with standard deviations or medians with interquartile ranges (IQRs). Categorical variables were expressed as counts and percentages. Paired comparisons between baseline and post-treatment values were performed using one-tailed paired *t*-tests, with the alternative hypothesis that post-treatment values would be lower than baseline. Statistical significance was defined as α = 0.05.

To identify independent predictors of FIB-4 change, a multivariable linear regression model was fit with ΔFIB-4 (FIB-4_post_ − FIB-4_pre_) as the continuous outcome variable. Predictor variables included change scores for BMI (ΔBMI), AST (ΔAST), ALT (ΔALT), and hemoglobin A1c (ΔA1c); baseline FIB-4 index; age; sex; presence of type 2 diabetes; total GLP-1 RA treatment duration in days; and GLP-1 RA agent (coded as dummy variables with semaglutide as the reference category). Negative ΔFIB-4 values indicate improvement. The model was estimated by ordinary least squares (OLS) with complete-case analysis (n = 27; two patients were excluded from this model due to missing A1c values). Multicollinearity was assessed using the variance inflation factor (VIF); predictors with VIF > 10 were noted. Standardized beta coefficients (Std β) were computed by refitting the model after scaling all continuous predictors to a mean of 0 and standard deviation of 1, enabling comparison of effect magnitudes across variables with different units. A reduced model incorporating the primary clinically prioritized predictors—ΔBMI, ΔA1c, baseline FIB-4, age, sex, and diabetes status—was also fit to assess BMI-independence of FIB-4 change with greater statistical power.

Analyses were conducted using the “stats” package in R version 4.1.3 (R Foundation for Statistical Computing, Vienna, Austria); multivariable linear regression was performed using the lm() function, with VIF calculated using the “car” package.

### Ethical considerations

The study protocol received approval from the Tufts Health Sciences Institutional Review Board (IRB ID: STUDY00005100), which, due to the retrospective nature of the research, waived the requirement for informed consent. All data were extracted from the EHR by authorized study personnel and de-identified before analysis.

## Results

### Baseline patient characteristics

Among 229 patients with MASLD who received GLP-1 RA therapy during the study period, 29 (12.7%) met inclusion criteria of a baseline FIB-4 index ≥ 1.30 and had sufficient follow-up data available in the EHR (Table 1). Of the 200 excluded patients, 89 (44.5%) did not have both baseline FIB-4 and post-treatment FIB-4 data available; 23 (11.5%) had no verifiable GLP-1 RA start date; 19 (9.5%) received GLP-1 RA therapy for less than 6 months at the time of analysis; and 69 (34.5%) had a baseline FIB-4 < 1.30.

**Table 1:** Baseline Patient Characteristics

Total (n)	29
Sex (n, %)	
Female	17 (58.6%)
Male	12 (41.4%)
Age (mean, median, IQR, range)	62.0, 60.9, 56.6–67.6, 40.8–79.3
BMI (mean, median, IQR, range)	37.5, 35.7, 32.0–41.7, 26.5–53.7
GLP-1 RA (n, %)	
Semaglutide	17 (58.6%)
Dulaglutide	8 (27.6%)
Liraglutide	2 (6.9%)
Tirzepatide	2 (6.9%)
GLP-1 RA indication (n, %)	
Type 2 diabetes	18 (62.1%)
Obesity	11 (37.9%)

BMI: body mass index; GLP-1 RA: glucagon-like peptide-1 receptor agonist; IQR: interquartile range.

At the start of GLP-1 RA therapy, the cohort of qualifying patients had a mean age of 62.0 years (median 60.9; IQR 56.6–67.6; range 40.8–79.3) and included 17 female patients (58.6%) and 12 male patients (41.4%). Mean baseline BMI was 37.5 kg/m^2^ (median 35.7; IQR 32.0–41.7; range 26.5–53.7). Semaglutide was the most frequently prescribed GLP-1 RA (n = 17; 58.6%), followed by dulaglutide (n = 8; 27.6%), liraglutide (n = 2; 6.9%), and tirzepatide (n = 2; 6.9%). Indications for therapy included type 2 diabetes in 18 patients (62.1%) and obesity in 11 patients (37.9%).

### Primary outcome

After 12 months of GLP-1 RA therapy, there was a statistically significant reduction in mean FIB-4 index from 1.94 to 1.73 (mean difference −0.21; 95% confidence interval (CI) −0.38 to −0.05; P = 0.019) (Table 2).

**Table 2:** Mean Values of Primary and Secondary Outcomes at Baseline and After 12 Months of GLP-1 RA Therapy

	Baseline (SD)	Post-GLP-1 RA (SD)	Difference (95% CI)	P-value
FIB-4	1.94 (0.61)	1.73 (0.62)	-0.21 (-0.38, -0.05)	0.019
AST (U/L)	48.9 (22.4)	33.1 (17.4)	-15.8 (-22.3, -9.3)	< 0.001
ALT (U/L)	60.3 (34.3)	38.4 (27.0)	-21.9 (-30.8, -13.0)	< 0.001
Platelets (× 10^3^/µL)	212.2 (51.1)	215.3 (57.6)	3.1 (-4.9, 11.1)	0.742
BMI (kg/m^2^)	37.5 (7.0)	34.9 (6.8)	-2.6 (-3.7, -1.5)	< 0.001
A1c (%)	7.4 (1.8)	6.3 (1.0)	-1.1 (-1.5, -0.6)	< 0.001

A1c: hemoglobin A1c; ALT: alanine aminotransferase; AST: aspartate aminotransferase; BMI: body mass index; CI: confidence interval; FIB-4: fibrosis-4; GLP-1 RA: glucagon-like peptide-1 receptor agonist; SD: standard deviation.

### Secondary outcomes

GLP-1 RA therapy was associated with significant improvements in liver enzyme levels and metabolic parameters (Table 2). Mean AST decreased from 48.9 to 33.1 U/L (mean difference −15.8 U/L; 95% CI −22.3 to −9.3; P < 0.001), and mean ALT declined from 60.3 to 38.4 U/L (mean difference −21.9 U/L; 95% CI −30.8 to −13.0; P < 0.001). Platelet counts showed no significant change (212.2 to 215.3 k/µL; mean difference 3.1; 95% CI −4.9 to 11.1; P = 0.742). In terms of metabolic parameters, mean BMI decreased from 37.5 to 34.9 kg/m^2^ (mean difference −2.6 kg/m^2^; 95% CI −3.7 to −1.5; P < 0.001), and mean A1c improved from 7.4% to 6.3% (mean difference −1.1%; 95% CI −1.5 to −0.6; P < 0.001).

### Stratification by GLP-1 RA agent

In descriptive analysis stratified by GLP-1 RA agent (Table 3), mean ΔFIB-4 was −0.19 for semaglutide (n = 17), −0.36 for dulaglutide (n = 8), −0.38 for tirzepatide (n = 2), and +0.38 for liraglutide (n = 2). Notably, the two liraglutide-treated patients had the highest mean baseline FIB-4 in the cohort (2.34). Given the small per-group sample sizes, formal hypothesis testing across agent groups was not performed.

**Table 3:** Change in FIB-4 Index (ΔFIB-4), BMI (ΔBMI), Hemoglobin A1c (ΔA1c), AST (ΔAST), and ALT (ΔALT) Stratified by GLP-1 RA Agent Over 12 Months

GLP-1 agent	n	Baseline FIB-4, mean (SD)	ΔFIB-4, mean (SD)	ΔBMI (kg/m^2^), mean (SD)	ΔA1c (%), mean (SD)	ΔAST (U/L), mean (SD)	ΔALT (U/L), mean (SD)
Semaglutide	17	1.84 (0.69)	−0.19 (0.55)	−2.72 (4.24)	−1.44 (1.48)	−16.2 (16.4)	−23.6 (26.9)
Dulaglutide	8	1.94 (0.51)	−0.36 (0.40)	−2.27 (2.01)	−0.59 (0.79)	−20.1 (20.7)	−26.8 (31.8)
Liraglutide	2	2.34 (0.04)	+0.38 (0.85)	−0.34 (3.04)	+0.50 (—)^b^	+14.5 (43.1)	+6.5 (38.9)
Tirzepatide	2	2.35 (0.52)	−0.38 (0.03)	−5.15 (2.11)	−0.60 (1.13)	−25.5 (26.2)	−17.0 (15.6)
Overall	29^a^	1.94 (0.61)	−0.21 (0.52)	−2.60 (3.56)	−1.05 (1.32)	−15.8 (21.3)	−21.9 (29.3)

Values are presented as mean (standard deviation). All delta values represent post-treatment minus baseline. ^a^n = 27 for ΔA1c (two patients with missing post-treatment A1c values excluded from that column only). ^b^SD not calculable for liraglutide ΔA1c; only one patient had complete A1c data. ALT: alanine aminotransferase; AST: aspartate aminotransferase; BMI: body mass index; FIB-4: fibrosis-4; GLP-1 RA: glucagon-like peptide-1 receptor agonist; SD: standard deviation.

### Multivariable analysis

To examine independent predictors of FIB-4 change, a multivariable linear regression model was fit in 27 patients with complete data (Table 4). The full model—incorporating ΔBMI, ΔAST, ΔALT, ΔA1c, baseline FIB-4, age, sex, diabetes status, GLP-1 RA treatment duration, and GLP-1 agent type—explained 89.1% of variance in ΔFIB-4. Six predictors reached statistical significance (P < 0.05).

**Table 4:** Multivariable Linear Regression: Independent Predictors of Change in FIB-4 Index (ΔFIB-4) Over 12 Months of GLP-1 RA Therapy (n = 27)

Variable	β	SE	95% CI	t	P-value	Std β
Intercept	−0.327	0.603	−1.621 to 0.966	−0.543	0.596	—
ΔBMI (kg/m^2^)	0.020	0.017	−0.015 to 0.056	1.219	0.243	0.074
ΔAST (U/L)	0.005	0.007	−0.011 to 0.021	0.662	0.519	0.104
ΔALT (U/L)	0.001	0.005	−0.009 to 0.010	0.182	0.858	0.024
ΔA1c (%)	−0.167	0.074	−0.324 to −0.009	−2.271	0.040*	−0.220
Baseline FIB-4	−0.538	0.121	−0.797 to −0.278	−4.446	< 0.001*	−0.334
Age (years)	0.024	0.011	0.001 to 0.046	2.243	0.042*	0.212
Male sex	0.519	0.138	0.222 to 0.816	3.749	0.002*	0.260
Diabetes mellitus	−0.649	0.223	−1.128 to −0.170	−2.906	0.012*	−0.312
GLP-1 duration (days)	−0.001	0.001	−0.002 to 0.001	−1.145	0.271	−0.083
Tirzepatide vs. semaglutide	0.870	0.291	0.245 to 1.494	2.987	0.010*	0.232
Liraglutide vs. semaglutide	1.250	0.489	0.202 to 2.298	2.559	0.023*	0.241
Dulaglutide vs. semaglutide	0.177	0.168	−0.184 to 0.538	1.052	0.311	0.082

Reference category for GLP-1 agent type: semaglutide. *P < 0.05. Full model: R^2^ = 0.891, Adjusted R^2^ = 0.798. Reduced model (ΔBMI, ΔA1c, baseline FIB-4, age, sex, DM; n = 27): R^2^ = 0.617, Adjusted R^2^ = 0.502, F(6,20) = 5.37, P = 0.002. ALT: alanine aminotransferase; AST: aspartate aminotransferase; BMI: body mass index; CI: confidence interval; FIB-4: fibrosis-4; GLP-1 RA: glucagon-like peptide-1 receptor agonist; SE: standard error; Std β: standardized beta coefficient (continuous predictors scaled to SD = 1).

Baseline FIB-4 index was the strongest significant predictor of FIB-4 change (β = −0.538, 95% CI −0.797 to −0.278; P < 0.001; Std β = −0.334), with higher baseline values independently associated with greater reduction. Although ΔA1c reached statistical significance in the full model (β = −0.167; P = 0.040), its adjusted coefficient was opposite in direction to its bivariate association with ΔFIB-4 (Pearson r = +0.27). Critically, change in BMI was not a significant predictor of ΔFIB-4 after adjusting for other covariates (ΔBMI: β = 0.020, 95% CI −0.015 to 0.056; P = 0.243).

Additional significant predictors in the full model included older age (β = 0.024; P = 0.042), male sex (β = 0.519; P = 0.002), and presence of diabetes mellitus (β = −0.649; P = 0.012). ΔAST (P = 0.519) and ΔALT (P = 0.858) were not independently significant; both variables demonstrated high collinearity (VIF = 11.0 and 7.6, respectively). Regarding GLP-1 agent type, with semaglutide as the reference, tirzepatide (β = 0.870; P = 0.010) and liraglutide (β = 1.250; P = 0.023) were associated with smaller reductions in FIB-4; dulaglutide did not differ significantly from semaglutide (β = 0.177; P = 0.311). The number of patients receiving tirzepatide (n = 2) and liraglutide (n = 2) was limited.

In the reduced model—restricted to ΔBMI, ΔA1c, baseline FIB-4, age, sex, and diabetes status (n = 27)—ΔBMI remained non-significant (β = 0.030; P = 0.174), further corroborating the independence of FIB-4 improvement from weight change (adjusted R^2^ = 0.502; F(6,20) = 5.37; P = 0.002). Baseline FIB-4 (β = −0.387; P = 0.010) and male sex (β = 0.594; P = 0.001) remained independently significant in the reduced model.

## Discussion

In this retrospective cohort study, 12 months of GLP-1 RA therapy in patients with MASLD and baseline risk of fibrosis (FIB-4 ≥ 1.3) was associated with a significant reduction in FIB-4 index. Improvements were also observed in AST, ALT, BMI, and A1c, suggesting concurrent benefits in hepatic inflammation and metabolic function.

Multivariable regression analysis identified several independent predictors of FIB-4 improvement (Table 4). The strongest predictor was baseline FIB-4 index (β = −0.538; P < 0.001), which may reflect regression to the mean in a high-risk population. None of the metabolic change variables, including ΔBMI (P = 0.243), ΔAST (P = 0.519), ΔALT (P = 0.858), or ΔA1c, were reliable independent predictors of ΔFIB-4; ΔA1c (P = 0.040) showed an opposite coefficient direction compared with its bivariate association, consistent with a suppressor effect among correlated predictors. Importantly, change in BMI was not an independent predictor of ΔFIB-4 in either the full (P = 0.243) or reduced model (P = 0.174) after covariate adjustment, suggesting that mechanisms beyond weight loss, such as a direct hepatoprotective effect, may contribute to the observed FIB-4 reduction. Patients with diabetes mellitus at baseline showed greater FIB-4 reduction (β = −0.649; P = 0.012), which may reflect greater metabolic responsiveness to GLP-1 RA therapy in this subgroup, though this observation warrants confirmation in larger cohorts.

Prior studies have highlighted the therapeutic potential of GLP-1 RAs in MASLD. For instance, a recent randomized, placebo-controlled study by Harrison et al demonstrated that treatment with pemvidutide, a GLP-1/glucagon dual receptor agonist, led to reductions in hepatic fat, markers of inflammation, and body weight over 12 weeks compared with placebo [18]. Similarly, clinical trials of semaglutide and tirzepatide have shown resolution of steatohepatitis without worsening of fibrosis. In a phase 2 trial, Newsome et al reported that semaglutide improved rates of NASH resolution but did not significantly change fibrosis stage over 72 weeks [14]. The SYNERGY-NASH phase 2 trial further demonstrated that tirzepatide increased rates of MASH resolution compared to placebo over 52 weeks, though without significant fibrosis regression [25].

More recently, evidence has begun to suggest that GLP-1 RAs may also influence fibrosis progression. The LEAN trial showed that liraglutide use over 48 weeks was associated with greater resolution of NASH (39% of patients receiving liraglutide versus 9% of patients receiving placebo, P = 0.019) and reduced risk of fibrosis progression (9% of patients receiving liraglutide versus 36% of patients receiving placebo, P = 0.04) [26]. Preliminary findings from the ongoing ESSENCE phase 3 trial demonstrated a higher rate of fibrosis reduction without worsening of steatohepatitis among patients treated with semaglutide compared with placebo (36.8% of patients receiving semaglutide versus 22.4% of patients receiving placebo, P < 0.001) [27]. These emerging data support the possibility of an antifibrotic effect, which aligns with the reductions in FIB-4 index observed in our study.

The identification of reliable noninvasive biomarkers remains a critical need as new pharmacotherapies for MASLD become available, including GLP-1 RAs and resmetirom [28, 29]. The FIB-4 index is an appealing contender given its ease of calculation and widespread use. Our findings, together with data from ESSENCE and other recent trials, suggest that FIB-4 may serve as a useful surrogate marker of fibrosis response to treatment.

Past studies evaluating FIB-4 changes with GLP-1 RA therapy have shown mixed results. Morieri et al reported no significant FIB-4 reduction in a large retrospective cohort of patients with type 2 diabetes, though not all had established MASLD or baseline fibrosis [30]. In a randomized control trial involving patients with type 2 diabetes and NAFLD, Liu et al found a modest, statistically significant reduction in FIB-4 with exenatide but not with insulin glargine, although there was no significant difference in FIB-4 change between the two groups, and baseline FIB-4 averaged around 1.0, below the typical threshold for fibrosis risk [31]. In a prospective cohort study, Tan et al demonstrated decreases in FIB-4 and other noninvasive fibrosis markers among patients with diabetes receiving liraglutide, though baseline scores were again close to 1.0 [32]. Our study adds to this literature by specifically focusing on patients with MASLD and FIB-4 ≥ 1.3, representing a population at intermediate or higher risk of fibrosis.

Interestingly, because age is a component of the numerator in the FIB-4 calculation, the index increases with time in the absence of treatment effect. The fact that FIB-4 decreased by a mean of 0.21 over 12 months in our cohort, during which all patients aged by 1 year, strengthens the inference that the observed reduction reflects a genuine biologic improvement in hepatic enzyme levels rather than a statistical artifact. It should be noted that FIB-4 is a mathematical composite of four variables, and reductions in this index reflect the net effect of changes across its components rather than a single discrete biological shift. In our cohort, platelet counts did not change significantly over the study period (+3.1 k/µL; P = 0.742), indicating that the FIB-4 improvement was driven primarily by reductions in AST and ALT, both of which declined significantly (AST −15.8 U/L and ALT −21.9 U/L, respectively; both P < 0.001). While these enzyme reductions are consistent with improved hepatic inflammation and reduced hepatocellular injury, they do not in isolation confirm structural fibrosis regression. The multivariable regression analysis provides additional granularity by examining the independent contribution of each metabolic parameter to FIB-4 change, though histologic validation remains the gold standard for confirming antifibrotic effects.

The clinical meaningfulness of a mean ΔFIB-4 of −0.21 warrants further investigation. While no universally accepted minimal clinically important difference (MCID) for FIB-4 has been established in the context of pharmacotherapy, this magnitude of change corresponded to a shift in risk strata for several patients in our study. Among the cohort, a subset of seven patients (24.1%) moved from the intermediate or higher risk group (FIB-4 ≥ 1.3) to the low-risk group (FIB-4 < 1.3), suggesting potential clinical reclassification. Future studies correlating ΔFIB-4 with biopsy-confirmed fibrosis stage change and long-term outcomes will be essential to establish a validated MCID for this index as a treatment endpoint in MASLD.

Strengths of this study include the focus on patients with MASLD and baseline evidence of fibrosis risk, use of real-world data across a large health system, and consistent improvements in multiple clinical and biochemical parameters. To our knowledge, this is the first study to specifically evaluate GLP-1 RA therapy in patients with MASLD and elevated baseline FIB-4. A major limitation of this study is the sample size: only 13% of the initially identified patients with MASLD and GLP-1 RA exposure met the FIB-4 inclusion criterion. Additionally, due to challenges in identifying a well-matched control group with MASLD who were not on GLP-1 RA therapy, this data is presented without a control group and is therefore limited in its ability to separate GLP-1 RA therapy from natural disease progression or concurrent lifestyle interventions. The absence of histologic data also precludes definitive conclusions about fibrosis stage at baseline or the histopathologic significance of observed FIB-4 changes.

Future research should assess FIB-4 alongside other noninvasive markers in larger, prospective cohorts, ideally with paired histology, to validate its utility as a surrogate endpoint for antifibrotic response. Studies comparing the magnitude of FIB-4 change with histologic regression will be essential to establish its role in clinical trials and routine practice. In parallel, incorporating complementary structural imaging modalities—such as vibration-controlled transient elastography (FibroScan), which provides direct noninvasive measurement of liver stiffness—into prospective study designs would enable cross-validation of FIB-4-based assessments with objective structural data, thereby strengthening confidence in FIB-4 as a practical surrogate endpoint.

Furthermore, it will be important to investigate whether specific GLP-1 RAs or dual agonists exert greater antifibrotic effects and whether patient subgroups, such as those with obesity, diabetes, and/or cardiovascular disease, derive differential benefit. Multivariable analysis in this study suggested that tirzepatide and liraglutide were associated with significantly less FIB-4 improvement compared to semaglutide in the adjusted model (tirzepatide: β = 0.870, P = 0.010; liraglutide: β = 1.250, P = 0.023). However, these findings must be interpreted with considerable caution: only two patients in this cohort received tirzepatide and two received liraglutide, rendering individual coefficient estimates highly susceptible to the influence of single observations. These exploratory findings nonetheless highlight the importance of adequately powered, head-to-head comparative studies of GLP-1 RAs and dual agonists, including tirzepatide, in patients with MASLD and established fibrosis risk.

Beyond their hepatic effects, GLP-1 RAs are increasingly recognized as pleiotropic cardiometabolic agents with broad organ-protective potential. Through attenuation of systemic inflammation, improvement of endothelial function, and reduction of visceral adiposity, GLP-1 RAs have been associated with reductions in major adverse cardiovascular events, heart failure hospitalizations, and progression of chronic kidney disease—benefits that are at least partially independent of glycemic control alone [33, 34]. In the context of MASLD, where cardiovascular disease is the leading cause of mortality and where hepatic, renal, and cardiac pathology frequently coexist [8], the hepatoprotective effects observed in this study may represent one component of a broader beneficial cardiometabolic phenotype. Prospective studies examining multi-organ endpoints in patients receiving GLP-1 RA therapy for MASLD—including cardiovascular event rates, renal function trajectories, and measures of arterial stiffness alongside hepatic biomarkers—would be valuable to characterize the full scope of benefit in this population [33–36].

In summary, in patients with MASLD at intermediate or higher risk of fibrosis, GLP-1 RA therapy was associated with significant reductions in FIB-4 index, aminotransferases, BMI, and A1c over 12 months, supporting a potential antifibrotic effect. The FIB-4 index may serve as a practical noninvasive marker for monitoring fibrosis response in MASLD, though further validation is required.

## Data Availability

Any inquiries regarding data availability for this study should be directed to the corresponding author.
